# Editorial: Regulation of pollen tube growth, volume II

**DOI:** 10.3389/fpls.2023.1242416

**Published:** 2023-07-11

**Authors:** Stefano Del Duca, Delia Fernández-González, Giampiero Cai

**Affiliations:** ^1^ Department of Biological, Geological and Environmental Sciences, University of Bologna, Bologna, Italy; ^2^ Department of Biodiversity and Environmental Management, University of León, León, Spain; ^3^ Department of Life Sciences, University of Siena, Siena, Italy

**Keywords:** fertilization, plant reproduction, pollen, cell-to-cell communication, environmental stress

The pollen tube is an extension produced by the pollen grain when conditions are favorable; thus, the pollen tube is important in seed plant reproduction because it transports male gametes. However, it is also an excellent system for studying various plant cell processes that are common to sink organs or tissues (Kroeger and Geitmann, 2012). The pollen tube has been used to study a variety of processes, including vesicular transport, cytoskeletal organization, cell wall deposition, ion gradients, intracellular signaling. Since the pollen tube grows by contacting and signaling to pistil cells, it is also a model for studying cell-cell communication (Broz and Bedinger, 2021). Moreover, the pollen tube is involved in self-incompatibility (SI) processes that regulate reproduction and thus promote hybridization and genetic variability (Mandrone et al., 2019). SI is regulated by several factors, and in some cases, such as citrus, it is an important tool for producing seedless mandarins (Gentile et al., 2012). Pollen tube and pollen can also be targets of environmental stresses (Ledesma and Sugiyama, 2019), which can impair plant reproductive success, resulting in lower productivity of agronomically important plants and increasing allergenicity (pollinosis) (Armentia et al., 2019; Singh and Mathur, 2021).

New information about the pollen tube is being published on a regular basis allowing us to better understand how the pollen tube promotes plant reproduction; the data also provides insight into how plant cells can change shape in response to specific external signals; and the data help us select plant genotypes that are more resistant to environmental stresses (Liu et al., 2006). For these reasons, we have agreed to serve as guest editors for this Research Topic on pollen tube growth regulation, which is a continuation of a previous one and covers a wide range of subjects.

A frequently asked question is whether the pollen tube nucleus is necessary for pollen tube growth. Motomura et al. used enucleated cells to show that the pollen tube can grow in the absence of its nucleus. This raises several concerns about the presence of persistent transcripts and thus the relative stability of previously produced mRNAs. Furthermore, this article emphasizes the independence of the pollen tube from the vegetative nucleus, at least with respect to growth.

Several colleagues discussed cell-to-cell communication. In their review, Bordeleau et al. summarized the known aspects of pollen-pistil communication in the terminal tract, but also highlighted the lack of knowledge about early pollen-pistil communication. Another review by Yu et al. focused on the role of peptides and receptors in pollen-pistil communication, a relatively unknown system of plant cell communication. The authors discussed the role of peptides in pollen tube growth and interaction with pistils, as well as communication between sperm cells and egg/central cells of the embryo sac. Serrano et al. also addressed the topic of cell-to-cell communication in their article. They focused on the lipidomic aspect of pollen and pollen tubes, examining changes in the levels of specific lipids during pollen tube growth. They found increasing levels of phosphatidic acid during cell growth, suggesting that proper cell-to-cell communication may require extensive use of these specific lipid components. Suanno et al. published another article on cell-cell communication, this time looking at a different and lesser-known pathway. The authors studied the production of small extracellular vesicles known as pollensomes and their possible role in the pollen-pistil communication mechanism. They found that extracellular vesicles can only be produced by germinated pollen and that they contain the ALIX protein, which is a known marker of extracellular vesicles in other cell systems. The manuscript helps us understand a new, unexplored mode of communication.

In their article, Seitz et al. studied the energy aspect of the pollen tube. They looked at the expression of sucrose transporters in pollen and found a gene (AtSUC1) that is critical for the pollen tube to accumulate the disaccharide. At the same time, they looked at genes for cell wall invertases that hydrolyze extracellular sucrose and allow the pollen tube to accumulate glucose *via* monosaccharide transporters. This is an important study because it advances our understanding of the energy dependence of the pollen tube on the pistil.

The attraction of the pollen tube by the synergid cells is clearly a priority before the fusion of the nuclei. Adhikari et al. examined the expression of MYB98 in synergid cells and found that this gene is important for guiding the pollen tube to contact the egg cell. The authors investigated the gene structure by identifying a cis-regulatory sequence and other genes that may target the above sequence. After the pollen tube is attracted, the next step is the fusion of sperm cells with egg cell and central cell, which is a fascinating topic that is unfortunately only partially understood; Sugi et al. investigated the removal of the inner vegetative plasma membrane (IVPM) of sperm cells prior to fusion and found that membrane disruption is required, emphasizing the importance of proper lipid composition. The mechanisms that control cell fusion are also unknown; González-Gutiérrez et al. described the presence of actin bundles that connect the central cell nucleus to the micropyle and may play a role in guiding male sperm cells to fuse. This aspect is only sporadically addressed in plant reproductive biology due to technical difficulties, but it sheds important light on the final step of the fertilization process. Indeed, the final stage of fertilization (e.g., the contact between pollen, egg cell, and synergids, as well as the fusion of the sperm nuclei with those of the female gametophyte) is undoubtedly less well understood.

Finally, Zhang et al. investigated the effects of environmental stress, focusing on the toxic effects of excess boron, by analyzing the composition of pollen tube cell walls. The cell wall is often the first target of contaminants because it is the outermost component. The authors have shown how excess boron alters the distribution of cell wall components, resulting in abnormal deposition of polysaccharides at the pollen tube tip; as a result, pollen tube growth is inhibited, leading to reduced reproductive success.

## Author contributions

SDD, DF-G, and GC contributed equally to the preparation and revision of the manuscript and approved the submitted version.
